# Extrusion of Polymer Nanocomposites with Graphene and Graphene Derivative Nanofillers: An Overview of Recent Developments

**DOI:** 10.3390/ma13030549

**Published:** 2020-01-23

**Authors:** José Sanes, Cristian Sánchez, Ramón Pamies, María-Dolores Avilés, María-Dolores Bermúdez

**Affiliations:** Grupo de Ciencia de Materiales e Ingeniería Metalúrgica, Campus de la Muralla del Mar., Universidad Politécnica de Cartagena, 30202 Cartagena, Spain; cristian.sanchez@upct.es (C.S.); ramon.pamies@upct.es (R.P.); mdolores.aviles@upct.es (M.-D.A.); mdolores.bermudez@upct.es (M.-D.B.)

**Keywords:** thermoplastics, graphene, graphene oxide, extrusion, nanocomposites

## Abstract

This review is focused on the recent developments of nanocomposite materials that combine a thermoplastic matrix with different forms of graphene or graphene oxide nanofillers. In all cases, the manufacturing method of the composite materials has been melt-processing, in particular, twin-screw extrusion, which can then be followed by injection molding. The advantages of this processing route with respect to other alternative methods will be highlighted. The results point to an increasing interest in biodegradable matrices such as polylactic acid (PLA) and graphene oxide or reduced graphene oxide, rather than graphene. The reasons for this will also be discussed.

## 1. Introduction

The fabrication of nanocomposites by the incorporation of nanofillers into polymer matrices originates materials with new functional properties while the excellent processing and manufacturing abilities are retained due to the flexibility in polymer matrices [1]. In recent years, the addition of different types of graphene (G) nanofillers has been attempted as a method to improve the mechanical and functional properties of a variety of thermoplastic matrices [2,3]. Graphene-based composites have demonstrated potential for applications in several technological fields such as energy storage [4,5], environmental applications [6] and biomedical usage [7,8,9].

Industrial thermoplastic polymers and composites are usually melt-processed by extrusion followed by injection molding. The extrusion step combines the application of high temperature and high specific mechanical energy in a short time interval. The optimization of extrusion parameters plays a fundamental role on the final properties of the material. Extrusion elements configuration and parameters such as residence time, screw speed, temperature and energy are even more critical when nanocomposites are concerned [10,11]. The final objective is to improve nanophase dispersion.

The original definition of graphene [12] is that of a single layer bidimensional structure of carbon atoms; however, the literature includes a few different carbon nanofillers under the common denomination of graphene [13].

Methods for achieving the dispersion of graphene in polymer matrices [14] usually include the use of a solvent in which both graphene and the polymer are dispersed and then co-precipitated [15]. Alternatively, the solvent is evaporated, and the dried dispersion is obtained as a film [16,17]. Another method that consists of adding graphene to the monomer before polymerization is carried out to obtain the corresponding composite material [18,19]. The following are the three main methods applied in obtaining polymer-graphene nanocomposites:

- Solvent processing. The use of conventional solvents causes serious health and environmental problems which prevent the large-scale usage of these procedures.
- In situ polymerization. This is an effective method to obtain small quantities of materials for laboratory research applications but not for industrial scale.
- Melt blending. Several thermoplastic matrices have been modified by melt blending addition of different forms of graphene oxide (GO) and reduced graphene oxide (rGO) nanofillers. However, this process might cause defects in the GO structure, from wrinkles and rolling, to modification of the initial aspect ratio.

Methods using a solvent need the previous modification of graphene surface in order to disperse it. In situ polymerization can be more efficient. However, both methods are not readily scaled to industrial processing. In contrast with solvent dispersion and in-situ polymerization processes, melt processing, that is, the direct dispersion of graphene nanophases into molten polymer is a cleaner process that can be readily scaled up for industrial applications. However, in order to achieve uniform graphene dispersion into highly viscous polymers, it is still necessary to modify the surface of graphene flakes. Although this modification can be made by chemical functionalization, with the formation of new covalent bonds [20], it can be more convenient to use a milder process involving non-covalent surface modification [21]. The ease of preparation of large amounts of graphene oxide, the relatively simple dispersion into polymer matrices and the affinity of GO surface oxygen-containing functional groups towards some macromolecules has made it the nanofiller of choice in many cases.

Melt-processing is usually performed by twin-screw extrusion, which is an environmentally-friendly method immediately applicable to the industrial scale manufacturing of final parts. The versatility of this process allows tailoring the extrusion parameters for each material and application. This method is widely used for the processing of nanocomposites, since the mixing action is based on an elongational flow. Shear and elongation stresses are as the material is transported through the space between screws (typically of the order of a few micrometers) [22].

Another advantage of extrusion could be the alignment of planar nanostructures such as those of graphene nanomaterials in one preferential direction. Kim et al. [23] have discussed the degree of alignment in polycarbonate-graphene nanocomposites as a function of manufacturing process. Nevertheless, the control and reproducibility of dispersion and orientation of GO inside polymer matrices are complex technical problems which, in most cases, remain to be solved [24]. In order to overcome the Van der Waals forces between graphene layers with an interaction energy of about 2 eV/nm^2^, the order of magnitude of the force necessary to exfoliate the graphite is about 300 nN/mm^2^ [25,26,27], which is very difficult to achieve.

Research interest in nanocomposite materials containing graphene has been present for more than a decade [28], as it begun very shortly afterwards the first description of graphene. The problems associated with the preparation and stability of graphene dispersions for final applications have been the object of previous reviews [26,27,28,29,30]. This review reports a brief overview on the recent research dealing with the formulation of polymer-based nanocomposites containing graphene or graphene oxide. In all cases, the manufacturing method of the reviewed composite materials has been melt-processing, in particular, twin-screw extrusion, which can then be followed by injection molding. Especial interest is devoted to the thermoplastic graphene nanocomposites melt-processed by extrusion and also to the biodegradable and biocompatible polymers.

## 2. Graphenic Nanofillers

**Figure 1:**
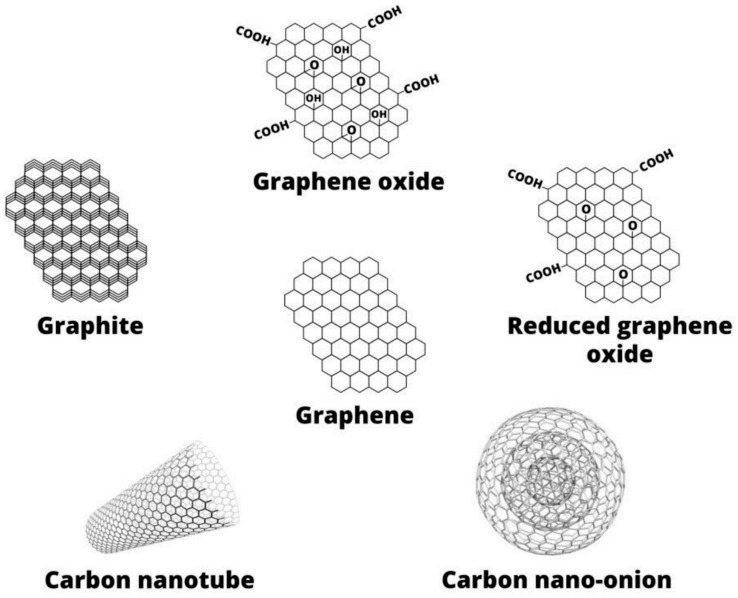
Structures of the most common graphenic nanofillers [38].

As it is mentioned above, there are several kinds of carbon nanofiller that are commonly called graphene. The basic structure of the different graphenic nanomaterials is graphene [31]. This nanomaterial is described as planar sheet of one-atom thickness, composed by carbon atoms with sp^2^ hybridization. As it is depicted in Figure 1, these carbon atoms are displayed into a honeycomb lattice. The carbon atoms are bound by means of two single and one double covalent bond to the three adjoining atoms [32,33]. The graphitic structure consists in multi-layered graphene sheets, and it is considered that G can be found in different arrangements, such as: graphene monolayers, graphene nanosheets and graphene nanoplatelets (GnPs) [34,35]. This nanomaterial exhibits remarkable attributes such as mechanical, electrical and thermal properties, among others, which are summarized in Table 1 [36,37,38].

One of the most widely used graphene derivatives is graphene oxide. The structure of this nanomaterial consists in graphene sheets with stoppages of sp^3^ hybrid carbon atoms with functional groups such as hydroxyl and epoxy groups in the surface and carboxylic groups at the edges [39,40,41]. Therefore, GO presents higher hydrophilicity than G and it can be better dispersed in aqueous media. This feature favors the possibility of functionalization of graphene-like materials. The chemical reduction of these functional groups results in reduced graphene oxide, which presents a similar structure than G. rGO shows higher electrical conductivity compared to GO and is more hydrophobic due to the low content of functional groups [42,43]. Other graphene-based nanostructures are carbon nanotubes (CNTs). Single-walled CNTs are formed by graphene sheets that are rolled into a cylindrical arrangement [44,45,46]. If the CNTs are composed by several layers, a structure consisted in concentric cylinders is formed and is commonly called multi-walled CNTs (MWCNTs).

## 3. Thermoplastic Nanocomposites

In this section we have summarized the recent publications on thermoplastic matrix composites modified by addition of graphene-derived nanofillers, modified graphene nanofillers or blends with other nanomaterials. These new nanocomposites have been obtained by extrusion, in most cases twin-screw extrusion, which can be followed by injection molding.

### 3.1. Polypropylene Matrix Nanocomposites

Table 2 shows that polypropylene (PP) matrix has been most commonly used [47,48,49,50,51,52,53,54,55,56,57,58,59,60,61,62] due to its availability and ease of processing. The effect of the concentration of nanofillers is a key parameter in the processing of nanocomposites. Iniesta et al. [47] added reduced graphene oxide in a range of concentrations between 0.05 and 1 wt. %, using twin screw extrusion, to improve mechanical properties such as tensile strength and hardness with respect to PP. Nevertheless, the formation of agglomerates of rGO nanoparticles was observed. The effect of variable concentration of GnP added to PP has been described [48], finding an optimum concentration of 0.4 wt. % for maximum mechanical resistance and a linear correlation between the melt shear viscosity and GNPs weight fraction was found. Extrusion processing of PP + G nanocomposites has been compared with surface coating and compression molding [49]. Graphene segregation took place under extrusion conditions; however, a continuous graphene network, with a percolation threshold of 0.4 wt. % was obtained by compression molding, thus increasing electrical conductivity. In order to avoid agglomeration of the nanofiller, elongation flow processing for the dispersion of GO sheets in isotactic PP has been employed [50]. The more effective dispersion of the nanophases led to the increase of the thermal stability and mechanical properties of the nanocomposites with increasing GO content.

The effect of graphene nanoplatelets (GnP) size on PP nanocomposites processed by twin-screw extrusion and injection molding has been studied [51] to find that smaller size nanofillers are the most effective in improving tensile strength and thermal stabilities, due to their lower tendency to agglomeration. These findings agree with computational studies [63]. On the other hand, large size nanofillers reduce the percolation threshold and increase electrical conductivity [52]. The role of large GnP as nucleating agents for PP crystallization has been also observed by Jun et al. [53] in extruded and injected nanocomposites. These observations are in agreement with the beneficial effect of large size GnP on the improvement of electrical conductivity but not on mechanical properties.

Recently, the combination of several nanofillers has demonstrated a useful strategy to obtain new nanocomposites with special properties. Synergy between graphene (G) and synthetic graphite (SG) was studied by Altay et al. [54], when added to PP matrix in a twin screw co-rotating extrusion machine. Mechanical properties and thermal stability were improved for high loads of G and SG. When GO is coated with short carbon fibers (SCF) [55], the extruded and injected PP-matrix nanocomposites showed higher thermal stability and mechanical resistance, which were attributed to the nucleating effect of GO-coated SCF for the crystallization of PP. Other researchers have combined graphene nanoplatelets (GnP) with the addition of other additives to PP matrices, such as large mass percentages of magnesium oxide and hydroxide and ammonium phosphate [56]. The new quaternary nanocomposites combined combustion resistance with high thermal conductivity.

In any case, agglomeration is an impasse in the development of PP-based nanocomposites [57]. During extrusion, rupture and erosion provoke that large GnPs agglomerates tend to be exfoliated into smaller platelets. He et al. [58] determined that the residence time is a key parameter for a better dispersion of GnP. Only erosion mechanism thin layers can be exfoliated and by increasing the residence time in a twin-screw configuration, the homogeneity of the particle size distribution and the dispersibility of the nanophase are enhanced. The resulting nanocomposites present higher electrical and thermal conductivities. Re-extrusion also improves the dispersion of GnPs in PP matrices; however, the crystal phases of the thermoplastic material are affected [59]. Hopmann and Adamy [60] have recently proposed a two-stage process. A solvent is used in the first step, and then, graphene is predispersed by ultrasonication, before adding the dispersion to the twin-screw extruder, where the solvent is removed. The improved graphene dispersion leads to nanocomposite materials with up to 57% increase of the elongation at break. The effect of ultrasonication during twin screw compounding of PP + GnP nanocomposites has been compared with that on PP + carbon nanotubes and PP + carbon black nanocomposites [61]. The results showed that the size of the agglomerates can be reduced by ultrasonication for other carbon nanophases but not for PP + GnP, thus showing the difficulty of processing graphene nanocomposites. Functionalized graphene is an effective strategy to improve the dispersion of the nanophase in the polymeric matrix. Functional properties of PP such as flame retardancy (FR) have also been improved by addition of different forms of carbon nanoadditives. Hoffmann et al. [62] not only achieved this improvement in PP-FR modified by thermally reduced graphene oxide (TRGO) and multilayer graphene (MLG), but they have also described a remarkable (80%) stiffness increase, combined with an increase of electrical conductivity. The best performance of TRGO is attributed to the presence of wrinkled functionalized graphene (FG) containing functional groups which enhance its dispersibility in the polymer matrix under extrusion conditions. The hydrophobicity of the nanophase can be tuned, and a better interaction with the polymer occurs during the melt extrusion process. Therefore, the electrical and mechanical properties of the nanocomposites are enhanced.

### 3.2. Other Thermoplastic Matrices

Although PP and PLA (see below) are the most widely studied matrices for graphene nanocomposites, many other thermoplastic materials have also been modified to obtain graphene nanocomposites (Table 3) [21,64,65,66,67,68,69,70,71,72,73,74,75,76,77,78,79,80,81,82,83,84,85,86,87,88,89,90,91,92,93,94].

Polyethylene (PE) and different types of polyethylene-derived materials such as linear low-density polyethylene (LLDPE), high density polyethylene (HDPE), or polyfluorinated ethylene propylene and polyethylene naphthalate (PEN) have been studied as matrices [64,65,66,67,68,69]. Reactive-melt extrusion of PE + G showed increasing crystallinity of the nanocomposites due to the nucleating effect of graphene. This increased not only mechanical properties but also the barrier to oxygen function of the polyolefin [64]. The mechanical tests on extruded and injected LLDPE-GO nanocomposites [65] showed an increase in tensile resistance, rigidity and hardness. In LLDPE-G nanocomposites, the effect of extrusion variables such as extruder and feeder speed has been studied [66,67]. One set of single-screw extrusion conditions was selected for HDPE [68], where the effect of graphene concentration, between 1 and 3 wt. % was studied. Polymers with biomedical applications such as hybrid HDPE-ultrahigh molecular weight polyethylene (UHMWPE) [69] have been modified by amine surface functionalized GO, to improve not only elastic modulus and tensile strength, but also its resistance to biodegradation, thus making the new nanocomposite a possible candidate for bone tissue applications. Polyfluorinated ethylene propylene melt-spinning fibers [70] were reported to improve their chemical resistance by the addition of graphene in a low (0.3 wt. %) proportion. The inclusion of the fibers provokes an increase on the mechanical properties, reporting a Young’s Modulus 19.5% higher; and an improvement of the lipophilic behavior. Therefore, this kind of materials shows promising applications in oil-water separation. A modification of the conformation of PEN polymer chains due to graphene nanofiller has been described [71]. Consequently, the crystallization of PEN occurs with the addition of graphene at very low concentrations (0.01–0.1 wt. %). The distribution and dispersion of the nanophase were more homogeneous, and a satisfactory intercalation of the graphene layers was found without the usage of additives or additional modifications of the nanophase. This was attributed to π–π interactions between graphene platelets and naphthalene rings.

Polyamides (PA) are another commonly used base polymer for the processing of graphene containing nanocomposites [72,73,74,75,76,77]. PA6/PLA blends have been modified with variable GnP concentrations [72], achieving maximum thermal and mechanical resistance for the highest GnP ratio of 5 wt.%. Few layers graphene (FLG) has been added to PA6/Poly (butylene terephthalate)-block-poly (tetramethylene glycol) (PBT-PTMG) to obtain ternary nanocomposites [73], using both in situ polymerization and extrusion. By this method, a low FLG concentration of 0.5 wt. % was sufficient to increase impact, flexural strength and elongation at break. A different method, consisting of masterbatch dilution was employed by Rashmi et al. [74] to obtain PA11/GnP materials, with enhanced mechanical properties for 5 wt. % GnP content. An acceleration of the crystallization process of PA in the presence of G has been described for nanocomposites extruded under the same conditions as the unmodified polymer [75]. The functionalization of graphene has been proved to enhance the dispersibility into the PA matrix through reactive extrusion [76].

**Figure 2:**
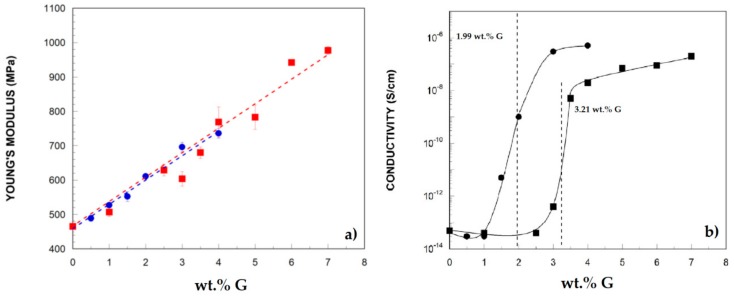
Increase of Young’s Modulus (**a**) and conductivity (**b**) of thermoplastic polyurethane (TPU) −based nanocomposites with the addition of graphene [78].

Other widely used thermoplastics include polystyrene (PS), thermoplastic polyurethane (TPU) or poly (methylmethacrylate) (PMMA) matrices [21,78,79]. Ionic liquids have been utilized to modify the surface of the nanophase aiming at better dispersibility in the polymeric matrix. In this case, the dispersion of IL-modified graphene resulted in a better dispersion of the nanophase. The obtained nanocomposites showed higher mechanical and electrical properties [78] (see Figure 2) and better processability [21]. Low concentrations of GO are used in PMMA matrices in presence of 1 wt.% TiO_2_ processed by using twin-screw extrusion [79] with potential dental applications. PA/PP composites [80] have been modified by functionalized GO, finding that GO concentrations should be maintained lower than 1 wt.% in order to maximize mechanical resistance. Liquid-phase feeding (LPF), and solid–solid deposition (SSD) have been proposed by Muñoz et al. [81] to reduce agglomeration of GO in order to scale the commercial processing of PS nanocomposites [82]. ABS (acrylonitrile butadiene styrene) has been reinforced by rGO [83], multi-walled carbon nanotubes or both nanofillers [84]. It has been proved that the addition of these nanophases also affects the rheological properties of the nanocomposites, increasing the elastic behavior with a reduction of the ductility. On the other hand, GnP have been used as additive in poly(carbonate) (PC) and PC-ABS blends [85,86], increasing the mechanical properties of the nanocomposite but without the loss of ductility and an improvement on the thermal stability. Reactive extrusion is utilized to prepare conductive nanocomposites composed by poly(butylenetherephtale) (PBT) with several graphenic materials [87].

Highly thermal, chemical and mechanical resistant polymers such as PSU (Polysulfone) [88], PPS (Polyphenylene sulfide) [89,90], PVDF (Polyvinylidene fluoride) [91,92] and PEEK (Polyether ketone) [93,94] have also been modified by rGO, GnP, GO or a combination of modified GO and modified SiO_2_. For example, the surface modification of graphene with PSU brushes increased the affinity of the nanophase with the thermoplastic matrix, resulting in the improvement of the nanophase dispersion. In the case of PVDF-GO composites, the addition of the nanophase leads to higher values of thermal and electrical conductivities. PEEK-based nanocomposites present an enhanced tribological performance, higher mechanical properties and improved electrical conductivity.

### 3.3. Biodegradable and Biocompatible Polymers

In the last century, plastics have become an integral part of our daily lives due to their excellent properties, such as density, mechanical properties, processability and price. However, plastic waste has changed into one of the most challenging problems in our society. The plastic waste production has been estimated in over 6 billion tons [95]. Currently, the production of plastic waste is approximately 300 million tons every year worldwide and only 10% of this waste is recycled [96,97]. Polylactic acid (PLA) is one of the most extensively used materials in functional products, as it is a biodegradable, non-contaminant material, exhibiting good mechanical properties that are frequently used in aerospace, automotive and biomedical engineering applications [98,99]. Therefore, the recent interest in the development of biodegradable materials has attracted increasing attention towards PLA nanocomposites [100]. Table 4 summarizes the very last PLA-graphenic composites prepared by means of melt blending [101,102,103,104,105,106,107,108,109,110,111] and other biodegradable and biocompatible polymers [112,113,114,115,116,117].

**Figure 3:**
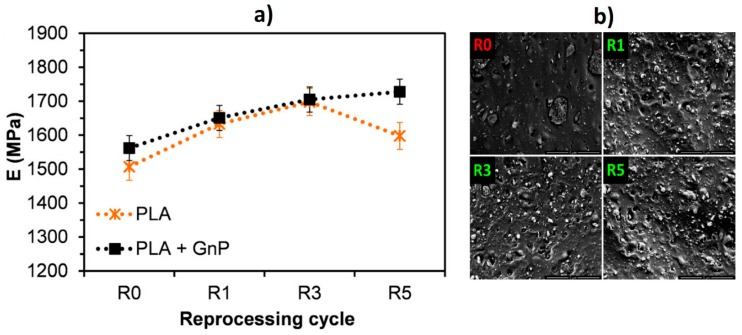
Variation of Young’s Modulus of polylactic acid (PLA) and PLA + graphene nanoplatelet (GnP) with the reprocessing cycles (**a**) and dispersion of GnP in the matrix (**b**) [104].

The versatility of extrusion processing is an advantage for the control of the microstructure. Thus, multilayer co-extrusion is used to orient graphene and obtain a layered material with alternating PLA and PLA + GnP layers [102]. In situ polycondensation of lactic acid has been used to obtain a masterbatch containing exfoliated graphene before melt extrusion of PLA and diluted masterbatch nanocomposite strips [103]. PLA-masterbatch with 0.05 wt. % graphene showed the best dispersion and mechanical properties. For recyclable materials, it is necessary to study the effect of reprocessing on their structure and properties. Interestingly, it has been described [104] that GnP decrease the degradation of PLA under repeated processing, using, in this case, a single screw extruder. Multiple extrusion cycles improved the dispersion of GnP in the polymer matrix, reducing the size of the aggregates (see Figure 3). In contrast, hydrolytic degradation of PLA under immersion conditions in a solution of NaOH was faster for PLA nanocomposites containing different nanophases, including graphene, than that of neat PLA [105].

The effect of the different types of nanofillers (graphene and graphene-CNTs blends) of PLA has been explored [108,109,110]. It has been found that the crystallinity of the nanocomposite was increased with the nanotube-containing. Extruded PLA films with 6 wt. % proportion of mixed GnP + carbon nanotube fillers incorporated by solution blending have been recently described [108]. The nanofillers were found to affect crystallinity, but the final properties of the materials depend on processing and ageing conditions. Increased crystallinity by combination of GnP and multiwalled carbon nanotubes has also been reported [109] for PLA. The reinforcing effect of the nanophases was confirmed by dynamic mechanical analysis (DMA). The functionalization of GO improves dispersion and increases crystallinity, thermal and mechanical properties, as it is discussed above with other thermoplastic matrices. A reactive extrusion method was employed to obtain PLA nanocomposite containing amidated graphene oxide [110].

Biodegradable nanocomposites have been obtained by different approaches, such as thermal reduction of GO during processing of PLA-PBS (polybutylene succinate)-rGO nanocomposites [111], and the uniform coating of PLA with graphene [106]. In this later case, not only the mechanical properties are improved but also the hydrophobicity of PLA. This is a relevant issue, as the affinity of PLA towards water is one of the major drawbacks in the use of this biodegradable matrix. Reactive blending of PLA with thermoplastic starch and GnP has been achieved by extrusion, and films were then obtained using cast-film extrusion [112]. In this case, the addition of GnP produces 900% toughness increase. A crack-bridging mechanism was proposed for the enhancement of mechanical properties, attributed to the high aspect ratio of the nanofiller.

In the case of the biodegradable and biocompatible plastics, different mixtures of graphene and other materials are used as fillers to preserve the sustainability of the composites. Manufacturing of biodegradable plastics can reduce the environmental impact and favor renewable resources. However, some properties of biodegradable polymers cannot often be comparable with petroleum-based plastics and the addition of fillers may enhance the physical, chemical and mechanical properties [114,115]. Biocarbon and GnP have been successfully incorporated to bio-based PBS (Poly (butylensucciante)), showing an exceptional increase of the mechanical properties when the dilution of the masterbatch is used instead of direct compounding method [116]. For PHBV (Poly (3-hydroxybutyrate-*co*-3-hydroxyvalerate)), the selected fillers are a combination of rGO and ZnO nanoparticles [117], to maintain the environmentally friendly characteristics of the base polymer.

## 4. Conclusions and Future Outlook

The present work is an overview on very recent developments in the field of graphene-thermoplastic nanocomposites melt processed by extrusion methods. A wide range of matrices have been studied, from commodity polymers to high resistant engineering polymers. Special attention is being paid at the development of biodegradable and biocompatible nanocomposites.

Extrusion is readily scalable to industrial manufacturing of final products; however, in most cases, obtaining homogeneous graphene dispersions within the polymer matrix is not a simple task. Segregation and agglomeration of the nanophases are responsible for the lack of improvement in the thermal, mechanical and electrical properties with respect to the unmodified matrix materials.

The results of this review denote that the most widely used techniques to improve the dispersion of graphenic nanofillers are sonication, surface modification and the application of a coating layer of graphene on the polymer surface. However, another promising technique that is beginning to yield positive results is the use of ionic liquids, which should be explored more thoroughly. Other strategies are focused on the modification of the extrusion process, the use of masterbatches and the combination of graphene with other materials as hybrid nanophases.

The main factors that affect the characteristics and attributes of these nanocomposites are the nanofiller size and concentration. It has been stated that larger sizes of nanofillers result in a reduction of the mechanical properties but an increase of other functional properties, such as electrical conductivity. However, the effect of graphene concentration is not so well defined, and more studies are necessary.

A relevant field, which has received limited consideration until the present moment, is that of the influence of the additives on the deformation and fracture modes and on the resistance to degradation of the final materials.

Finally, another line of work that is starting to receive attention is the tribological performance of extruded nanocomposites as a function of extrusion parameters, polymer structure, nanofiller orientation and surface modification.

## Figures and Tables

**Table 1:** Mechanical and functional properties of pristine graphene.

**Mechanical Properties**	
Young’s modulus	1 TPa
Fracture Strength	130 GPa
**Electrical Properties**	
Electrical conductivity	10^4^ S/cm
Electron mobility	250,000 cm^2^/V·s
**Other Properties**	
Thermal conductivity	5300 W/mK
Specific surface area	2630 m^2^/g
Optical transmittance	97.7%

**Table 2:** Polypropylene-graphene nanocomposites melt-processed by extrusion.

Matrix	Nanophase	Reference
PP (Polypropylene)	Reduced graphene oxide (rGO)	[47]
PP	GnP	[48,49,51,52,53,57,58,59,60]
PP	GO	[50]
PP	GnP + SG (synthetic graphite)	[54]
PP	GO + short carbon fibers	[55]
PP	GnP, MgO and others	[56]
PP	GnP + carbon nanotubes + carbon black	[61]
PP-FR (flame retardant polypropylene)	TRGO (thermally reduced GO) MLG (multilayer graphene)	[62]

**Table 3:** Thermoplastic graphene nanocomposites melt-processed by extrusion.

Matrix	Nanophase	Reference
PE (Polyethylene)	Graphene	[67]
LLDPE (Linear low density Polyethylene)	GO	[65]
LLDPE	GnP	[66,67]
HDPE	GnP	[68]
HDPE/UHMWPE (High density polyethylene/Ultrahigh molecular weight polyethylene)	GO	[69]
FEP (polyfluorinated ethylene-propylene)	Graphene	[70]
PEN (Polyethylene naphthalate)	GnP	[71]
PA6 (Polyamide 6)	GnP	[72]
PA6	Graphene + PBT-PTMG	[73]
PA11 (Polyamide 11)	GnP	[74]
PA	Graphene	[75]
PA6	Functionalized Graphene	[76]
PA	GO	[77]
TPU (Thermoplastic Polyurethane)	Graphene Graphene modified by ionic liquid	[78]
PMMA	GO GO modified by ionic liquid	[21]
PMMA (Polymethylmethacrylate)	GO + TiO_2_	[79]
PA/PP	GO	[80]
PS (Polystyrene)	GO	[81]
PBAT (Poly(3-hydroxybutyrate- *co*-3-hydroxyvalerate)		
PS	Graphene	[82]
ABS (Acrylonitrile butadiene styrene)	rGO and/or MWCNT	[83,84]
PC-ABS Polycarbonate-(acrylonitrile butadiene styrene)	Graphene nanoplatelets (GnP)	[85]
PC (Polycarbonate)	GnP	[86]
PBT (Poly (butylenetherephtalate)	GnP, GO and rGO	[87]
PSU (Polysulfone)	rGO	[88]
PPS (Polyphenylene sulfide)	GnP	[90]
PVDF (Polyvinylidene fluoride)	GO	[91,92]
PEEK (Polyether ketone)	Modified GO + modified SiO_2_	[93]
PEEK	GnP	[94]

**Table 4:** Biocompatible and biodegradable graphene nanocomposites melt-processed by extrusion.

Matrix	Nanophase	Reference
PLA Poly(lactic acid)	Graphene	[101,102,103,105,106]
PLA	GnP	[104]
PLA	GnP + CNT	[107,108,109]
PLA	Amidated graphene oxide	[110]
PLA/PBS (Polylactic acid/polybutylene succinate)	GO	[111]
Thermoplastic starch/PLA	Graphene	[112,113]
PHB (Poly3-hydroxybutyrate-*co*-3-hydroxybutyrate)	GnP + A-fnSiO_2_	[114]
PHB	GO Hydrophobically modified GO	[115]
PBS	GnP + Biocarbon	[116]
PHBV (Poly3-hydroxybutyrate-*co*-3-hydroxyvalerate)	rGO + ZnO	[117]
